# Effect of Intravenous Lidocaine on Rocuronium: A Randomized Controlled Trial

**DOI:** 10.24546/0100493127

**Published:** 2025-02-12

**Authors:** SAWAKO TAKEBE, SHINYA TAGUCHI, NORIHIKO OBATA, SATOSHI MIZOBUCHI

**Affiliations:** 1 Department of Anesthesiology, Kobe University Hospital, Kobe, Japan

**Keywords:** Rocuronium, Lidocaine, Onset time, Duration of action, Muscle relaxant

## Abstract

**PURPOSE:**

Under general anesthesia, lidocaine shortens the onset time of vecuronium but it does not affect that of rocuronium. We suspected that the dose of rocuronium used in previous reports, 0.6 mg/kg, was too high to detect any difference due to lidocaine’s effect. We investigated the effects of 1.5 mg/kg lidocaine on the onset time, 50% depression time, 100% depression time, and duration of action of 0.4 mg/kg rocuronium.

**METHODS:**

Sixty adult patients who underwent elective operation under general anesthesia without tracheal intubation were randomly divided into two groups: including a lidocaine group (LG) and a placebo group (PG). Anesthesia was induced and maintained using propofol and remifentanil. After the loss of consciousness, a supraglottic device was inserted. After the neuromuscular monitor was calibrated, measurements were initiated with train-of-four stimulation at 20-second intervals, and 1.5 mg/kg lidocaine and the same volume of physiological saline were administered to the LG and the PG respectively. Ten seconds after the next train-of-four stimulation, 0.4 mg/kg rocuronium was administered.

**RESULTS:**

The mean (standard deviation) onset time (seconds) between the LG and PG were 368.0 (170.5) and 314.8 (161.1), respectively, with no significant difference (*p* = 0.24). There were no significant differences between the groups in terms of the 50% depression time (*p* = 0.71), 100% depression time (*p* = 0.53), or duration of action (*p* = 0.45).

**CONCLUSION:**

The pre-administration of 1.5 mg/kg lidocaine did not affect the onset time, 50% depression time, 100% depression time, or duration of action of 0.4 mg/kg rocuronium.

## INTRODUCTION

Lidocaine has long been used as an antiarrhythmic agent and local anesthetic. It is often used during general anesthesia to prevent and treat ventricular arrhythmias associated with surgery as well as to reduce vascular pain associated with propofol, a general anesthetic, and rocuronium, a non-depolarizing muscle relaxant, especially in pediatric patients. Intravenous administration of local anesthetics enhances neuromuscular blockade produced by both depolarizing and non-depolarizing muscle relaxants [1, 2]. Therefore, when lidocaine is used during general anesthesia, the effect of concurrently administered muscle relaxants may be enhanced, resulting in a shorter onset time and a longer duration of action.

Three published clinical trials have studied the interactions between intravenous lidocaine and aminosteroid non-depolarizing muscle relaxants. One study investigated the effect of vecuronium and found that pre-administration with an intravenous bolus of 1.5 mg/kg lidocaine was shown to shorten the onset time of vecuronium, but the duration of action was not reported [3]. The remaining two studies investigated the effects of rocuronium. One study involved the administration of an intravenous bolus of 1.5 mg/kg lidocaine followed by continuous infusion of 2 mg/kg/h before 0.6 mg/kg rocuronium and found no impact on either the onset time or the duration of action of rocuronium [4]. The other study involved the administration of intravenous bolus of 1.5 mg/kg lidocaine before 0.6 mg/kg rocuronium, and found no impact on the onset time but partially prolonged the recovery times of rocuronium [5]. These studies suggest that lidocaine may shorten the onset time of vecuronium but does not affect that of rocuronium.

The effects of lidocaine on neuromuscular blockade were not clearly defined in these studies, as the results may differ depending on the study method. First, other drugs with neuromuscular-blocking effects (midazolam [4], lidocaine [4], and isoflurane [5]) were administered before the control value for neuromuscular monitoring was determined. Therefore, the reliability of the measurement results is questionable. Second, the dose of rocuronium was the intubation dose (0.6 mg/kg), and at this dose, the onset time of rocuronium was probably too short to detect any difference due to the effect of lidocaine. A small dose of rocuronium below the intubation dose was administered to control the airway using a supraglottic device or to prevent generalized convulsions associated with modified electroconvulsive therapy. However, no studies have been found that investigated the effects of lidocaine combined with a small dose of rocuronium.

To provide a basis for the safe use of lidocaine in these anesthesia methods, we investigated the effect of 1.5 mg/kg lidocaine on the onset time and the duration of action of 0.4 mg/kg rocuronium.

## MATERIALS AND METHODS

This study was designed as a randomized, double-blind, placebo-controlled, parallel-group trial performed at the Kobe University Hospital International Clinical Cancer Research Center.

We report this trial in accordance with the Consolidated Standards of Reporting Trials (CONSORT) guidelines. The trial was approved by the Ethics Committee at Kobe University Hospital (Kobe, Japan) on June 29, 2023 (A230001, -1). The protocol was registered with the University hospital Medical Information Network Clinical Trial Registry (UMIN000050673 on June 26, 2023) before patient enrollment in the trial began. All eligible and consenting patients provided their signed, written informed consent before randomization.

### Study population

Inclusion criteria included patients with American Society of Anesthesiologists (ASA) physical status of I or II, aged 50 years to under 80 years, scheduled for elective operation under general anesthesia without tracheal intubation.

Exclusion criteria included patients with alanine aminotransferase >100 U/L, creatinine >1.5 mg/dL, neuromuscular diseases, a history of multiple drug allergies, contraindications to lidocaine administration such as severe heart conduction system disorders or allergy to amide-type local anesthetics, receiving propranolol or amiodarone, and expected to have difficulty with airway management.

### Study design

#### Randomization

The allocation was performed using simple computerized randomization. Eligible and consenting patients were randomly assigned in a 1:1 ratio to either the Lidocaine group (LG) or the Placebo group (PG). The researcher not involved in anesthesia generated the random allocation sequence, enrolled participants, and assigned participants to interventions.

#### Blinding

The patient and anesthesiologist in charge of the patient were blinded to the allocation group. Lidocaine or saline (both colorless and transparent) was prepared in a syringe by an investigator who was different from the anesthesiologist. The allocation result and drug administration were not entered into the anesthesia record until neuromuscular monitoring was completed.

### Intervention

All patients were monitored by an electrocardiogram, non-invasive blood pressure, pulse oximetry, and a bispectral index monitoring system (BIS^TM^; Medtronic, Minneapolis, MN, USA). Anesthesia was induced with 0.1 μg/kg/min remifentanil and 4 μg/ml propofol using a target-controlled infusion system (Terufusion TCIpump TE-371^TM^; TERUMO, Tokyo, Japan). After the patient lost consciousness, a supraglottic device (i-gel^TM^; Intersurgical, Berkshire, UK) was inserted. Anesthesia was maintained using remifentanil and propofol, adjusted appropriately according to the blood pressure, heart rate, and bispectral index. The patient was ventilated with a target end-tidal carbon dioxide partial pressure (EtCO_2_) of 40–45 mmHg, as respiratory pH changes may affect the action of rocuronium [6, 7]. After the EtCO_2_ had stabilized within that range, the neuromuscular monitor was calibrated to determine the control value. The measurements with train-of-four (TOF) stimulation were initiated at 20-second intervals. Once a stable baseline measurement was obtained, a bolus dose of 1.5 mg/kg lidocaine or the same volume of 0.9% physiological saline was administered intravenously for 1 minute. After completing the administration of lidocaine or saline, 0.4 mg/kg rocuronium and a subsequent 10-ml saline flush were administered 10 seconds after the next TOF stimulation.

#### Neuromuscular monitoring

Neuromuscular monitoring and measurements were performed as recommended by the international consensus conference [8]. The neuromuscular function was assessed using electromyography (TetraGraph FTG2001^TM^; Senzime AB, Uppsala, Sweden) in the upper limb that did not have an infusion route. Surface electrodes (TetraSens^TM^; Senzime AB, Uppsala, Sweden) were applied to the cleaned skin, along with the ulnar nerve of the wrist. The round proximal recording electrode was placed over the abductor digiti minimi (ADM) muscle. The arm was secured to the armrest with a wide cloth to maintain it in the same position throughout the study procedure. The temperature of the distal end of the forearm was maintained at >32°C, and the axillary body temperature was maintained at >36°C using a patient warming system (WarmTouch™; Covidien Japan, Tokyo, Japan).

After the supraglottic device was inserted and the EtCO_2_ had stabilized within 40–45 mmHg, electromyography was calibrated by Auto mode and subsequent 2-Hz TOF stimulation every 20 seconds was started. All data recorded by electromyography were transferred to a computer using the software program (TetraConnect) and stored. We adopted the data measured by the Tetra Graph, truncated to the first decimal place.

### Outcomes

The primary outcome was the onset time (OT), which was the time in seconds from the administration of rocuronium to the maximum depression of the first twitch (T1) of the TOF responses [9]. For the OT, we adopted the first-time point at which the same minimum value was observed for two or more consecutive measurements.

**Fig. 1:**
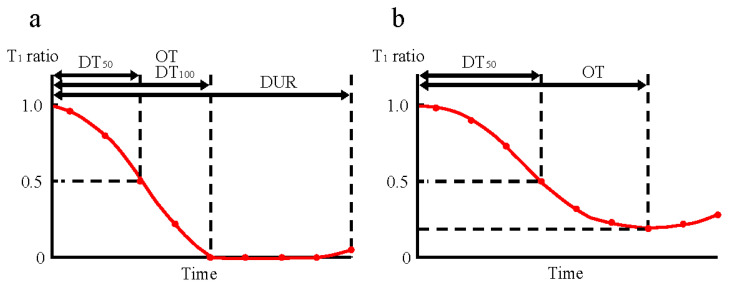
Outcome measurement methods The horizontal axis represents the time since the administration of rocuronium. The vertical axis represents the T_1_ ratio relative to the control value. DUR is the time from the administration of rocuronium to the T_1_ reappearance. a: When the minimum value of the T_1_ ratio reaches zero, OT, DT_50_, DT_100_, and DUR are measured. OT and DT_100_ are equivalent. b: When the minimum value of the T_1_ ratio does not reach zero, only OT and DT_50_ are measured. DT_100_ and DUR cannot be measured. T_1_, the first twitch of the TOF; OT, onset time; DT_50_, 50% depression time; DT_100_, 100% depression time; DUR, duration of action; TOF, train-of-four.

The secondary outcomes were 50% depression time (DT_50_), 100% depression time (DT_100_), and duration of action (DUR) (Fig. 1).

- DT_50_: the time in seconds from the administration of rocuronium to the first moment when the T_1_ value decreased to 50% or less of the control value. Cases in which the T_1_ did not decrease to 50% or less of the control value were excluded from the comparison.
- DT_100_: the time in seconds from the administration of rocuronium to 100% decrease in the T_1_ from the control value. We adopted the first-time point at which the same minimum value was observed for two or more consecutive measurements. Cases in which the T_1_ did not decrease to 100% of the control value were excluded from the comparison.
- DUR: the time in minutes from the administration of rocuronium to the T_1_ reappearance. We adopted the first-time point at which T_1_ reappeared for two or more consecutive measurements. Cases in which the T_1_ did not disappear were excluded from the comparison.

### Sample size and statistical analyses

A previous study examining the OT of 0.4 mg/kg rocuronium reported a mean of 155 seconds with a standard deviation (SD) of 40 seconds [10]. We determined that the clinically relevant OT-shortening effect of lidocaine was 20% (31 seconds). For the analysis of the primary outcome, a *t*-test with a two-sided significance level of 5% and a power of 80% would require 27 subjects per group. With an estimated dropout rate of approximately 10%, the target number of subjects for the study was set at 30 in each group, with a total of 60 subjects in the 2 groups.

#### Statistical method

Our statistical analysis plan was registered before unblinded data became available. Summary statistics (mean, SD, minimum, median, and maximum) were calculated for each group, and *t*-tests were used for comparisons between the two groups. If a normal distribution of the data could not be assumed, the Mann–Whitney *U* test was also performed. The significance level was set at 5% (two-sided), and a 95% confidence interval (two-sided) was calculated. All statistical analyses were performed using EZR version 1.61 (Saitama Medical Center, Jichi Medical University, Saitama, Japan), an R-based analysis software program [11].

## RESULTS

### Participants

**Fig. 2:**
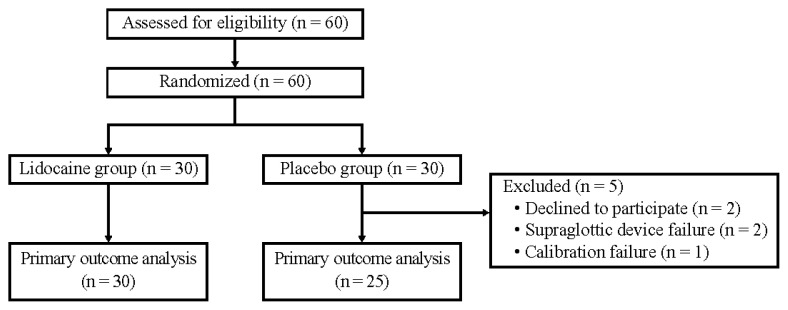
Study flow chart

Sixty patients were included in the study between August 2023 and March 2024, and 55 were included in the final analysis (Fig. 2). All patients met the inclusion criteria and did not meet any of the exclusion criteria. We randomly divided these 60 patients into the LG and PG. Of these, 5 patients dropped out of the study, and the protocol was finally conducted on 55 patients (30 in the LG and 25 in the PG) with primary outcome results.

The baseline characteristics are reported in Table I and Appendix 1. Patient characteristics were comparable between the groups.

### Primary outcome

**Fig. 3:**
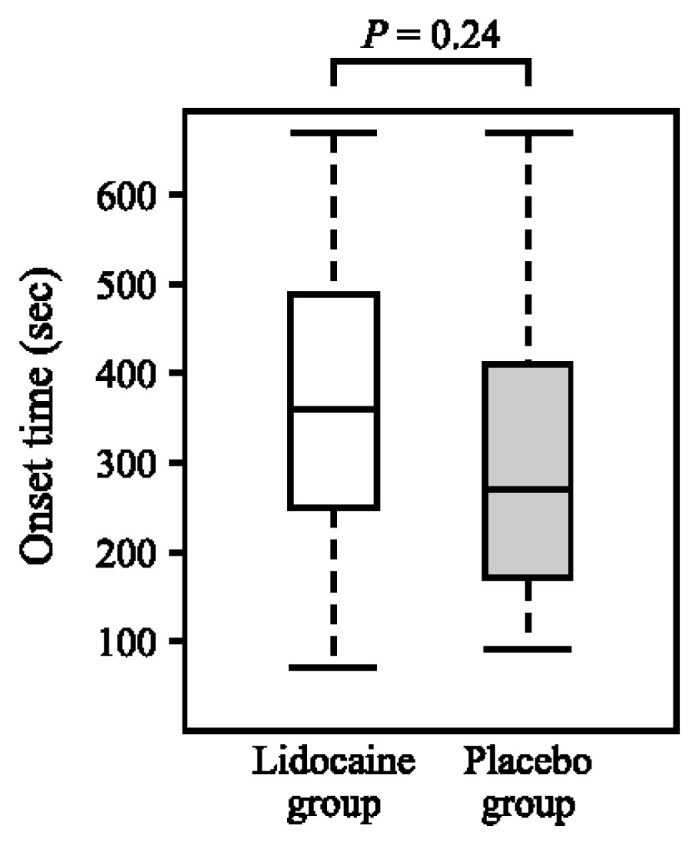
Box-and-whisker plot of onset time in Lidocaine and Placebo groups The time in seconds from the administration of rocuronium to maximum depression of the first twitch of the train-of-four responses. *P*-value was reported using Student’s *t*-test, and no statistically significant difference was found between the two groups. The box plot shows the median, interquartile range (IQR), and the range of values within 1.5 times the IQR. The line inside the box represents the median.

**OT**. The mean (SD) OT (seconds) for the LG (n = 30) and PG (n = 25) were 368.0 (170.5) and 314.8 (161.1), respectively, with no significant difference (*p* = 0.24) (Fig. 3, Table II, and Appendix 2).

### Secondary outcomes

**DT_50_**. The median [interquartile range] DT_50_ (seconds) for the LG (n = 30) and PG (n = 25) were 91 [75–160] and 90 [70–130], respectively, with no significant difference (*p* = 0.71).

**DT_100_**. The mean (SD) DT_100_ (seconds) for the LG (n = 13) and PG (n = 15) were 243.8 (149.8) and 215.4 (82.9), respectively, with no significant difference (*p* = 0.53).

**DUR**. The mean (SD) DUR (minutes) for the LG (n = 8) and PG (n = 12) were 17.9 (6.8) and 20.3 (6.8), respectively, with no significant difference (*p* = 0.45) (Table II and Appendix 2).

## DISCUSSION

Intravenous administration of local anesthetics enhances neuromuscular blockade produced by both depolarizing and non-depolarizing muscle relaxants [1, 2]. In fact, it has been reported that intravenous administration of lidocaine before vecuronium, a non-depolarizing muscle relaxant, shortens the OT [3], whereas administration before rocuronium, a non-depolarizing muscle relaxant, does not affect the OT [4, 5]. However, these previous studies had problems in that (i) other drugs with neuromuscular-blocking effects were administered before the control value for neuromuscular monitoring was determined, and (ii) the OT of rocuronium was probably too short to detect any difference due to the effect of lidocaine. This study, after addressing these issues, demonstrated with the highest accuracy to date that the administration of lidocaine as a premedication does not shorten the onset time of rocuronium. Moreover, the fact that the results remained unchanged even when using a different measurement method (electromyography) from previous studies further enhances the reliability of the present findings.

**Fig. 4:**
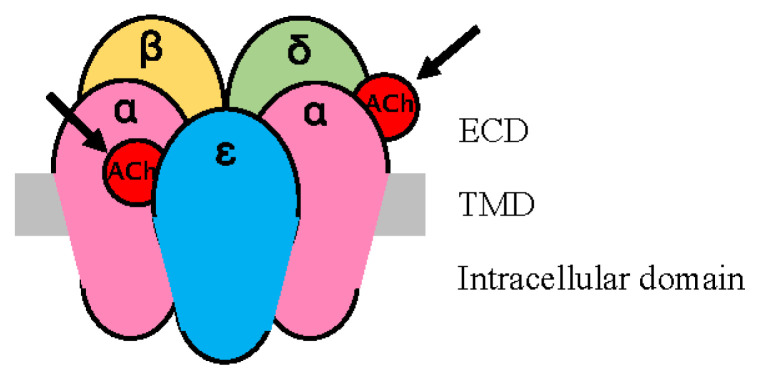
The nAChR and ACh binding sites The mature nAChR consists of five subunits. The ECD harbors ACh binding sites at α/ɛ and α/δ interfaces (indicated by arrows). The TMD forms a channel pore. nAChR, nicotinic acetylcholine receptor; ACh, acetylcholine; ECD, extracellular domain; TMD, transmembrane domain.

Although local anesthetics such as lidocaine primarily act as sodium channel blockers, lidocaine has also been shown to act on acetylcholine (ACh) receptors (AChRs) and reversibly block ACh-induced currents [12]. At small doses, local anesthetics suppress presynaptic ACh release [13], and at larger doses, they also inhibit postsynaptic AChRs and block ACh-induced muscular contractions [12, 14]. In AChR, the regions outside, surrounded by, and inside the cell membrane are called the extracellular domain (ECD), transmembrane domain (TMD), and intracellular domain, respectively (Fig. 4). Virtual docking assays using nicotinic AChR (nAChR) models revealed that lidocaine binds to the channel pore, TMD, and ECD [15]. In fact, in vitro experiments have shown that the local anesthetic benzocaine inhibits nAChR in various states (resting and active states), and a model is being considered in which local anesthetics act on TMD and ECD depending on the state of AChR [16]. As one of the putative binding sites for lidocaine in the ECD is the ACh binding site of the α subunit [15], it may compete with ACh molecules as well as muscle relaxant molecules.

To explain our results, we consider four possible mechanisms: (1) the effects of different speeds of presynaptic receptor inhibition, (2) the effects of different preferential binding sites on postsynaptic receptors, (3) the effects of different potencies of muscle relaxants, and (4) the effects of structural changes in nAChR.

### (1) Effects of different speeds of presynaptic receptor inhibition

Vecuronium and rocuronium have similar potency to presynaptic AChRs inhibition [17], but rocuronium inhibits them faster than vecuronium [18]. Local anesthetics also inhibit presynaptic AChRs and suppress ACh release [13], lidocaine may have accelerated the OT by compensating for the delay in vecuronium’s presynaptic inhibition.

### (2) Effects of different preferential binding sites on the postsynaptic receptors

The mature nAChR consists of five subunits (α, ɛ, α, δ, and β). It has two non-identical binding sites for ACh, located at the interface between the α and ɛ subunits and the interface between the α and δ subunits [19] (Fig. 4). It is known that some combinations of nAChR antagonists can act not only additively but also synergistically compared to when each is used alone. Waud et al. [20] proposed that additive or synergistic responses could be explained by assuming that muscle relaxants preferentially bind to one site or the other. They hypothesized that the synergistic effect of d-tubocurarine and pancuronium arises from their preferential binding to opposing interfaces. Indeed, it was later shown that d-tubocurarine preferentially binds to the α/ɛ interface [21], and pancuronium preferentially binds to the α/δ interface [22].

Vecuronium also preferentially binds to the α/δ interface, whereas rocuronium has no site selectivity [22]. Comparing the dissociation constants, the affinity of vecuronium to the α/δ interface is more than twice as high as that of rocuronium, whereas the affinity of vecuronium to the α/ɛ interface is approximately one-seventh that of rocuronium [22]. Lidocaine also probably has no site selectivity, since the ACh binding sites on the α subunit are predicted to be the main binding sites for lidocaine in the ECD [15]. Lidocaine is primarily a sodium channel blocker, and previous reports suggest that its affinity for AChRs is much lower than that of rocuronium [12, 22]. Therefore, for vecuronium, which has an affinity for one site, the combination with lidocaine, which has no site selectivity, acts synergistically because lidocaine binds relatively easily to the α/ɛ interface, a site of low affinity for vecuronium. On the other hand, the combination of rocuronium and lidocaine, both of which have no site selectivity, may produce additive or no effect because of the higher affinity of rocuronium than lidocaine at both sites. The dose of lidocaine used in this study (1.5 mg/kg) does not produce any clinical neuromuscular blockade, and therefore may not have influenced the effect of rocuronium.

### (3) Effects of different potencies of muscle relaxants

For a muscle relaxant to exert its action, the receptor occupancy rate must exceed the “margin of safety” [23]. In the case of vecuronium, pre-administration of lidocaine may have had an effect similar to the “priming principle” [24] of increasing receptor occupancy in advance. However, the potency of rocuronium is approximately one-sixth that of vecuronium [25], so the number of molecules of rocuronium administered clinically is approximately six times that of vecuronium (as the molecular weights of rocuronium and vecuronium are almost the same). All potent drugs that depend on binding to a certain population of receptors for their effect may have an OT that is modified by the number of available molecules compared with the total number of receptors [26], so the OT of rocuronium is shorter than that of vecuronium. In this study, the number of molecules administered as 0.4 mg/kg of rocuronium was approximately three times that of 0.15 mg/kg vecuronium in a previous study [3]. Therefore, it remains possible that the OT was too short to detect any effect of lidocaine.

### (4) Effects of structural changes in the nAChR

It has been suggested that conformational changes induced by antagonist binding may contribute to synergistic effects [19]. Recently, it has been reported that the general anesthetic etomidate binds to the desensitized state of nAChRs, potentially delaying their return to an activatable resting state, and increases the binding affinity of agonists to nAChRs [27]. Similarly, it is possible that prior administration of lidocaine caused a structural change in the receptor that favored vecuronium binding but did not affect rocuronium binding. As an example of a similar potential mechanism, it has been reported that the recovery times of vecuronium and pancuronium are prolonged by approximately 2 hours after suxamethonium administration compared to when they are used alone [28]. Given that approximately 90% of intravenously injected suxamethonium is metabolized within 1 minute [29], this phenomenon may also be due to suxamethonium causing a structural change in the nAChR that favors the binding of vecuronium and pancuronium, rather than an interaction of muscle relaxants. To verify this effect, the molecular structure and mechanism of AChR need to be elucidated in detail.

Several limitations associated with the present study warrant mention. First, our results may change if the protocol is changed, such as by increasing the lidocaine dose beyond 1.5 mg/kg. Regarding the timing of rocuronium administration, it has been shown that the blood concentration of lidocaine peaks immediately after administration [30], which is believed to not affect the results of this study. Other factors that may affect the OT include injection speed, cardiac output [9], muscle blood flow, and neuromuscular monitoring procedures [31]. However, because the patients were randomized, and anesthesia and neuromuscular monitoring methods were performed according to the same protocol, the influence of these factors was considered to be minimal. Second, the neuromuscular monitor used in this study was only measured at 20-second intervals, so subtle differences may not have been detected. Finally, previous studies on vecuronium have used acceleromyography of the adductor pollicis (AP) muscle, but this study used electromyography of the ADM muscle. Different muscles have different sensitivities to muscle relaxants; therefore, using different muscles and measurement methods may affect the results. It has been reported that although there is no significant difference in the OT between using an acceleromyography on the AP muscle and electromyography on the ADM muscle, there is a significant difference in the DUR [32]. Therefore, we believe that the difference in measurement methods did not affect the OT, which was the primary outcome; however, it may have affected the DUR.

In conclusion, the pre-administration of 1.5 mg/kg lidocaine did not affect the OT, DT_50_, DT_100_, or DUR of 0.4 mg/kg rocuronium.

## Figures and Tables

**Table I:** Patient demographic data

	Lidocaine group (n = 30)	Placebo group (n = 25)	*p*-value
**Female**	3/30 (10%)	3/25 (12%)	0.81
**Age (y)**	70.5 [63–75]	72.0 [67–77]	0.12
**Height (cm)**	167.0 (6.6)	168.0 [162.0–170.0]	0.65
**Weight (kg)**	65.6 (9.3)	69.3 (9.1)	0.24
**Surgery**			
**Prostate gold marker or spacer placement**	19/30 (63%)	13/25 (52%)	
**Prostate biopsy**	8/30 (26%)	9/25 (36%)	
**Mastectomy**	3/30 (10%)	2/25 (8%)	
**Orthopedic implant removal**	0/30 (0%)	1/25 (4%)	

Data are expressed as numbers (%) and mean (SD) or median [IQR]. SD, standard deviation; IQR, interquartile range.

**Table. II:** Results of primary and secondary outcomes of the study

Outcome	Lidocaine group		Placebo group		Mean difference [95%CI]		*p*-value
**OT (sec)**	n=30,	368.0 (170.5)	n=25,	314.8 (161.1)	53.2,	[−37.2 to 143.5]	0.24
**DT** **_50_** ** (sec)**	n=30,	91 [75–160]	n=25,	90 [70–130]	NA		0.71
**DT** **_100_** ** (sec)**	n=13,	243.8 (149.8)	n=15,	215.4 (82.9)	28.5,	[−63.9 to 120.8]	0.53
**DUR (min)**	n=8,	17.9 (6.8)	n=12,	20.3 (6.8)	−2.4,	[−8.9 to 4.1]	0.45

Data are expressed as numbers, mean (SD) or median [IQR], mean difference [95% CI], and *p*-value. We used Student’s *t*-test for parametric data (OT, DT_100_, and DUR) and the Mann–Whitney *U* test for nonparametric data (DT_50_). A *p*-value < 0.05 was considered significant.

OT, onset time; DT_50_, 50% depression time; DT_100_, 100% depression time; DUR, duration of action; CI, confidence interval; NA, not applicable; SD, standard deviation; IQR, interquartile range.

**Appendix 1:** Patient demographic data

	Lidocaine group		Placebo group	
**Age (y)**	68.6 (7.6),	70.5 [51, 78]	71.3 (7.1),	72.0 [52, 79]
**Height (cm)**	167.0 (6.6),	167.9 [151, 180]	165.6 (7.7),	168.0 [146, 176]
**Weight (kg)**	65.6 (9.3),	65.3 [45.8, 86.3]	69.3 (9.1),	70.0 [56.0, 85.1]

Data are expressed as mean (SD), median [minimum, maximum].

SD, standard deviation.

**Appendix 2:** Results of primary and secondary outcomes of the study

Outcome	Lidocaine group		Placebo group	
**OT (sec)**	368.0 (170.5),	360 [70, 670]	314.8 (161.1),	270 [90, 670]
**DT** **_50_** ** (sec)**	129.3 (85.4),	91 [50, 390]	111.6 (52.8),	90 [50, 230]
**DT** **_100_** ** (sec)**	243.8 (149.8),	230 [70, 630]	215.4 (82.9),	210 [90, 370]
**DUR (min)**	17.9 (6.8),	16.8 [9.8, 28.5]	20.3 (6.8),	19.3 [9.5, 32.5]

Data are expressed as mean (SD), median [minimum, maximum].

OT, onset time; DT_50_, 50% depression time; DT_100_, 100% depression time; DUR, duration of action; SD, standard deviation.
